# Federated learning improves site performance in multicenter deep learning without data sharing

**DOI:** 10.1093/jamia/ocaa341

**Published:** 2021-02-04

**Authors:** Karthik V Sarma, Stephanie Harmon, Thomas Sanford, Holger R Roth, Ziyue Xu, Jesse Tetreault, Daguang Xu, Mona G Flores, Alex G Raman, Rushikesh Kulkarni, Bradford J Wood, Peter L Choyke, Alan M Priester, Leonard S Marks, Steven S Raman, Dieter Enzmann, Baris Turkbey, William Speier, Corey W Arnold

**Affiliations:** 1 Department of Radiological Sciences, University of California, Los Angeles, Los Angeles, California, USA; 2 Department of Bioengineering, University of California, Los Angeles, Los Angeles, California, USA; 3 National Cancer Institute, National Institutes of Health, Bethesda, Maryland, USA; 4 Clinical Monitoring Research Program Directorate, Frederick National Laboratory for Cancer Research, Frederick, Maryland, USA; 5 Department of Urology, SUNY Upstate Medical Center, Syracuse, New York, USA; 6 NVIDIA Corporation, Bethesda, Maryland, USA; 7 Department of Urology, University of California, Los Angeles, Los Angeles, California, USA; 8 Department of Pathology and Laboratory Medicine University of California, Los Angeles, Los Angeles, California, USA

**Keywords:** deep learning, federated learning, privacy, generalizability, prostate

## Abstract

**Objective:**

To demonstrate enabling multi-institutional training without centralizing or sharing the underlying physical data via federated learning (FL).

**Materials and Methods:**

Deep learning models were trained at each participating institution using local clinical data, and an additional model was trained using FL across all of the institutions.

**Results:**

We found that the FL model exhibited superior performance and generalizability to the models trained at single institutions, with an overall performance level that was significantly better than that of any of the institutional models alone when evaluated on held-out test sets from each institution and an outside challenge dataset.

**Discussion:**

The power of FL was successfully demonstrated across 3 academic institutions while avoiding the privacy risk associated with the transfer and pooling of patient data.

**Conclusion:**

Federated learning is an effective methodology that merits further study to enable accelerated development of models across institutions, enabling greater generalizability in clinical use.

## INTRODUCTION

The disposition of healthcare data has generated significant interest in recent years. With the rapid expansion of the use of software-enhanced medical diagnostics, devices, and other interventions, access to clinical data has become critical to innovation. Clinicians and healthcare researchers facing this new data climate are forced to balance their profession’s ethical directives to “protect patient privacy in all settings to the greatest extent possible” and to “contribute to the advancement of knowledge and the welfare of society and future patients.”^1^ When the sharing of data is contemplated, ethics committees must evaluate the relative risks of unauthorized protected health information disclosure against the benefits of performing research and innovation using healthcare data.

An important contributor to the demand for healthcare data is the rapid advent of artificial intelligence (AI)-enhanced applications. For example, the field of medical image analysis has been driven forward in recent years by the advent of deep learning (DL). DL has enabled a wave of innovation in imaging decision support, with recent major results in the fields of ophthalmology,^2–4^ dermatology,^5–7^ pathology,^8–10^ and radiology.^11^^,^^12^

A major limitation of the DL approach is the need for a large volume of training data that captures the full breadth of inputs on which the model is likely to be subsequently used. In the field of natural image processing, large-scale pooled datasets with over a million images captured by a variety of different cameras are commonly used.^13^ This large volume is required because deep learning models are primarily *interpolators*, not *extrapolators—*that is, they perform best when presented with inputs that are similar to the data that those models were trained on. This creates the need to ensure that models intended for widespread clinical use are exposed to heterogeneous data sources that capture the full breadth of the patient populations, clinical protocols, and data acquisition devices (ie, scanners) that they will be used on.

However, medical imaging data in most cases is siloed within provider institutions, and, as a result, assembling large-scale datasets traditionally requires the transfer of data between these silos. Such transfers present ethical and legal challenges around preserving patient privacy. As a result, very few public large-scale pooled medical image datasets exist. This has led to a challenge of generalizability for DL models in medical imaging research, which are often trained on single-institution datasets. Such models often suffer from poor performance when transferred to other institutions with differing protocols, equipment, or patient populations.^14^^,^^15^ As a result, there is a need for methods to enable the development of general models for clinical use, without requiring the creation of pooled datasets.

An alternative methodology to centralizing multicenter datasets is known as “distributed” learning.^16^ In this paradigm, data are not combined into a single, pooled dataset. Instead, data at a variety of institutions are used to train the DL model by distributing the computational training operations across all sites. One such approach is federated learning (FL).^17–21^ In FL, models are trained simultaneously at each site and then periodically aggregated and redistributed. This approach requires only the transfer of learned model weights between institutions, thus eliminating the requirement to directly share data. However, a limitation of this approach is that no single model ever “sees” a complete picture of the universe of potential inputs during the training phase, thus placing pressure on the federated aggregation function to adequately distribute knowledge from each site into the model. Previous work has demonstrated the potential utility of FL for model training, generally using publicly available data to simulate multi-institutional training. However, works that examine the practical application of FL in radiological applications are still limited.^20^^,^^21^ Our work shows that FL can be reduced to practice using real-world private clinical data across multiple institutions, and that this approach creates a model that demonstrates improved generalizability both within the participating institutions and with outside data.

In this work, we demonstrate the application of FL at 3 institutions: University of California, Los Angeles (UCLA); the State University of New York (SUNY) Upstate Medical University; and the National Cancer Institute (NCI). For this demonstration, we used the medical image analysis task of whole prostate segmentation, an initial step for MRI diagnosis of cancer and fusion-guided interventions. We demonstrate that FL training and aggregation is able to produce a model that learns general predictive weights applicable to each institution dataset and demonstrates improved generalizability when applied to an external validation dataset.

## MATERIALS AND METHODS

### Study overview

**Figure 1.**
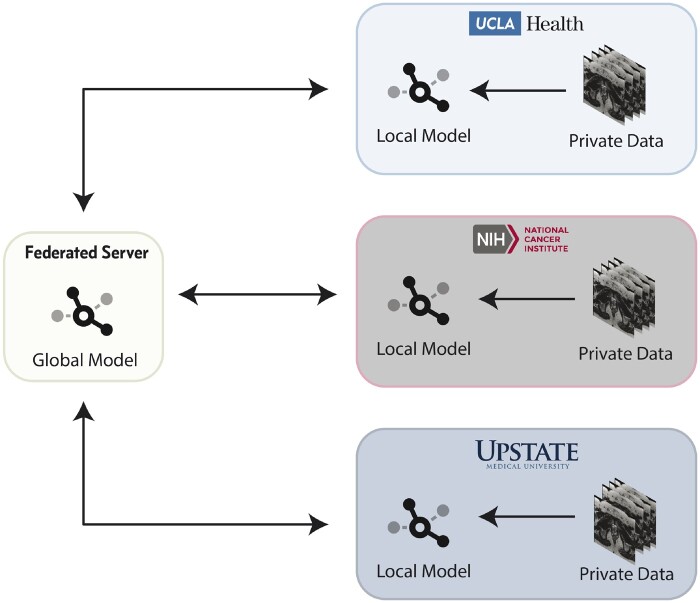
Federated learning architecture overview.

In this study, we use data collected retrospectively from each of our institutions to train and validate DL models to perform whole prostate segmentation on MRI. At no point during this study were private data transferred or shared across institutions. Instead, training on private data was done at the data’s respective institution, and model weights were iteratively aggregated by a federated server and redistributed (Figure 1). After training, we evaluated the generalizability of each of the models using held-out testing sets from each institution as well as an external challenge dataset.

### Data governance

One of the major challenges in multicenter DL studies is data governance. Our collaboration included 1 industry partner (nVIDIA, Inc.), 2 public universities (UCLA and SUNY Upstate), and 1 federal institution (NCI). For this study, UCLA, SUNY Upstate, and the NCI established a 2-way agreement with nVIDIA to collaborate and share model weights, but no material transfer agreement to exchange protected or private data was required. All 3 academic institutions had IRB approval for review and image analysis, with written informed patient consent or waiver of patient consent.

### Datasets and preprocessing

Each institution retrospectively collected 1 prostate MRI from each of a cohort of 100 patients enrolled in an IRB-approved protocol studying the use of MRI for prostate cancer diagnosis (the “private datasets”). Axial T2 weighted (T2W) images of the prostate acquired at 3T were obtained for each patient. A ground truth whole prostate segmentation was produced for each patient by an expert clinician at each institution (radiologist or urologist ranging from 9 to 27 years of experience). Segmentations were performed under the standard manual and semi-automatic clinical methodologies in place at the individual institutions. In order to demonstrate broad generalizability, participating institutions intentionally made no effort to harmonize either the T2W acquisition protocol or segmentation methodologies. In addition, 343 axial T2W images of the prostate were obtained from the public SPIE-AAPM-NCI PROSTATEx dataset^22^ (the “challenge dataset”). These images were annotated with ground truth whole prostate segmentations by an expert clinician.

Each T2W image and annotation included in the study was resampled to an isotropic 1mm x 1mm x 1mm voxel size. The images were then converted to the NIFTI format^23^ for training, and the intensity values within each image were normalized to zero mean and unit variance. Each of the private datasets was divided into a training set of 80 images and a held-out test set of 20 images.

### Model architecture and data augmentation

The 3D Anisotropic Hybrid Network^24^ (3D AH-Net) was used as the DL model for this study. The training metric was the soft Dice loss, and the Adam optimizer with validation metric-based learning rate decay was used for training. Real-time data augmentation was performed using the Deep Stacked Transformation^25^ methodology with random cropping based on the background/foreground ratio.

### Training strategy and federated model aggregation

Each private training set of 80 images was split into 5 sets of 16 images each. Then, for each experiment, 5 submodels were trained, each using 1 of the sets of 16 images as validation data, and the remainder as training data. The resulting submodels were then combined into a single ensemble model outputting the mean of all 5 submodels. The same cross-validation training sets were used for all experiments. A total of 4 training experiments were performed: 1 training run to develop a private model at each institution and an additional training run to develop an FL model across all institutions.

All models were trained for 300 epochs. For the FL training, a cloud-based federated weight aggregation server (“federated server”) was deployed by UCLA on a secure Amazon Web Services instance using the Clara application framework (nVIDIA, Inc.). Bilateral websocket connections (over Secure Sockets Layer/Transport Layer Security encryption) were established during training between each institution’s training server and the cloud-based aggregation server. After each training epoch, model weights and validation metrics from each institution for that epoch were sent to the server, where an aggregation function^26^ was used to combine them into a single set of model weights which were then sent back to each institution. These weights were then used as the basis for the next training epoch, and the process was repeated until all epochs had elapsed. The aggregation function used a weighted average of input models to produce the combined model. Each institution’s input was weighted based on the validation metric (mean Dice coefficient) from the most recent training epoch reported by the corresponding institution on the validation set for that fold. The FL training framework was implemented using the nVIDIA Clara Train SDK,^27^ and training at each site was performed using single nVIDIA GPUs.

### Statistical analysis

Each of the ensemble models was evaluated at each institution using its held-out test set, producing an evaluation for each model at each institution. In addition, each of the models was also evaluated on the challenge dataset. The evaluation metric used to compare segmented volumes was the Dice coefficient function as denoted in Equation 1, where $S_{DL}$ is the segmentation of a deep learning model and $S_{m}$ is the manual segmentation. The value of the coefficient can range between 0 (no overlap) and 1 (perfect overlap).

(1) $$\mathrm{Dic}e\;\left(S_{DL},S_{m}\right)=\frac{2|S_{DL\;}\cap \;S_{m}|}{\left|S_{DL}|+|S_{m}\right|}$$

The mean Dice coefficient was then compared for each model on each of the individual private test sets as well as the overall mean Dice coefficient for each model (across all of the test set data). The mean Dice coefficient was also separately computed for each model on the challenge dataset. Finally, 2-sided paired *t*-tests were used to compare the mean Dice coefficients from each private model to the FL model, for both the “combined” private test set and the held-out challenge dataset.

All data was used for this work under the approval of the appropriate institutional review board (UCLA IRB# 16-001087, SUNY IRB# 1519142-1, NCI IRB# NCI-18-C-0017).

## RESULTS

Patient and imaging characteristics of the 3 private datasets are shown in Tables 1 and 2. Tables 3 and 4 show all experimental results. The private models performed well on their own private test sets (Dice coefficient range: 0.883–0.925) but had diminished performance on the other private test sets (Dice coefficient range: 0.575–0.887). This led to overall mean Dice coefficients between 0.745 and 0.833 for the private models.

**Table 1.** Patient demographics

		Private Test Set Institution		
		NCI	SUNY	UCLA
Patient demographics	Age (years)	66 (47–83)	66 (49–81)	65 (50–83)
	Prostate size (cc)	65.5 (21.7–231)	72.9 (26.8–210)	52.1 (15.8–147)

**Table 2.** Image acquisition parameters

	Private Test Set Institution			
	NCI			
	with endorectal coil (n = 50)	without endorectal coil (n = 50)	SUNY	UCLA
Vendor(s)	Philips Medical Systems		Siemens	Siemens
Field strength	3T		3T	3T
In-plane resolution (mm)	0.273mm	0.352mm	0.625mm	0.664mm
Slice thickness (mm)	3mm	3mm	3mm	1.5mm
Repetition Time (TR, ms)	4775	3686	5500	2230
Echo Time (TE, ms)	120	120	136	204

**Table 3.** Model evaluation results—private test sets

		Private Test Set Institution			
		NCI (*n *=* *20)	SUNY (*n *=* *20)	UCLA (*n *=* *20)	**Overall** (*n *=* *60)
Private models	NCI	0.925 ± 0.016	0.854 ± 0.050	0.720 ± 0.165	0.833 ± 0.131*
	SUNY	0.887 ± 0.027	0.906 ± 0.018	0.768 ± 0.064	0.854 ± 0.074*
	UCLA	0.777 ± 0.102	0.575 ± 0.177	0.883 ± 0.069	0.745 ± 0.178*
FL Model		0.920 ± 0.029	0.880 ± 0.034	0.885 ± 0.032	0.895 ± 0.036

* Significantly lower than FL model (*P* < .001).

**Table 4.** Model evaluation results—ProstateX challenge dataset

		ProstateX (*n *=* *343)
Private models	NCI	0.872 ± 0.062*
	SUNY	0.838 ± 0.043*
	UCLA	0.812 ± 0.136*
FL Model		**0.889 **±** **0.036

* Significantly lower than FL model (*P* < .001).

In comparison, the FL model performed well on all 3 test sets. The FL model exhibited private test set mean Dice coefficients between 0.880 and 0.920, yielding an overall result of 0.895. The statistical analysis using 2-sided paired *t*-tests demonstrated that the FL model was significantly superior to any of the private models (*P *<* *.001 for all comparisons).

The private models exhibited varied performance on the challenge dataset (Dice coefficient range: 0.812–0.872). The generic FL model outperformed each of the private models, with an overall mean Dice coefficient of 0.889. The statistical analysis again demonstrated that the FL model was significantly superior to any of the private models (*P *<* *.001).

## DISCUSSION

We sought to demonstrate that data-distributed learning can be successfully operationalized across multiple institutions with real patient data using federated learning, and that the resulting model would gain the benefit of having learned from each of the private datasets without ever needing to transfer or pool data at a single location.

Since no transfer of protected health information (or even deidentified health information) was required, we were able to address the privacy and data governance limitations inherent to multicenter studies through the use of simplified 2-way collaboration agreements, rather than requiring the negotiation of a complex 4-way collaboration and material transfer agreement that would have been required if data were shared across institutions. This allowed for expedited ethics and compliance reviews because of the minimal risk posed by the FL paradigm and enabled us to be assured that our patients’ privacy was maintained.

The FL model that we trained performed well across all of the private datasets, yielding an overall performance level that was significantly better than that of any of the private models alone. This suggests that the FL model was able to benefit from the advantage of learning important institution-specific knowledge through the FL aggregation paradigm, without requiring any individual training site to “see” the full breadth of inputs.

Additionally, our results showed that the FL model performed significantly better than any of the individual private models on the held-out challenge dataset, suggesting that the model also attained the expected advantages inherent in training with more data through the FL aggregation method, even though the full dataset was not seen at any single training site.

Our work does have limitations. In this work, we did not attempt to address the potential for an inside actor (ie, 1 of the participating institutions) to attempt to recover the underlying patient data through a model inversion attack on the trained weights shared during federated learning. Future enhancements to the federated approach could include the addition of calibrated distortion to shared model weights in order to suppress the potential for inversion. However, we believe the method we demonstrate in this paper significantly better protects the privacy of patients than the current standard of direct sharing of data. In addition, though model inversion is a technical risk that cannot be ruled out, we empirically believe that the practical risk of inversion outside of crafted malintent on the part of study designers to be low due to the weight averaging scheme in place. Finally, we note that the sharing of trained model weights is an accepted practice within healthcare,^28^^,^^29^ and, in the worst case, our method is no less secure as only model weights are ever transmitted.

Secondly, the task we used (prostate segmentation on T2-weighted MRI) is relatively simple and all private models achieved high performance on their own institutional datasets. Thus, we were unable to demonstrate the expected benefit that an FL-trained model would significantly outperform a single-site-trained model on that single site’s data. In addition, because we used similarly sized private datasets at each institution, we did not explore the potential in varying the federated model aggregation methodology, which could be extended to differentially weight model weights from institutions based on data quantity, quality, or other metrics. Thirdly, adding additional institutions to the federation may present new challenges in heterogeneity of imaging data quality, governance, intellectual property, and model generalizability. In order to ensure that the FL model performs well at each institution in a large federation, it may be necessary in future work to explore adding an additional private fine-tuning step at each institution, though care must be taken to avoid losing generalizability through overfitting. This may require the use of additional techniques, such as the Learning without Forgetting method.^30^

## CONCLUSION

The power of federated learning was successfully demonstrated across 3 academic institutions using real clinical prostate imaging data. The federated model demonstrated improved performance across both held-out test sets from each institution and an external test set, validating the FL paradigm. This methodology could be applied to a wide variety of DL applications in medical image analysis and merits further study to enable accelerated development of DL models across institutions, enabling greater generalizability in clinical use.

## FUNDING

This work was supported by NIH NCI grant/contract numbers F30CA210329, R21CA220352, P50CA092131, ZIDBC011242, Z1ACL040015, HHSN261200800001E, NIH NIGMS grant number GM08042, NIH grant numbers, the AMA Foundation, an NVIDIA Corporation Academic Hardware Grant, the NIH Center for Interventional Oncology, the Intramural Research Program of the NIH, and a cooperative research and development agreement (CRADA) between NIH and nVIDIA.

The content of this publication does not necessarily reflect the views or policies of the Department of Health and Human Services, nor does mention of trade names, commercial products, or organizations imply endorsement by the US Government.

## AUTHOR CONTRIBUTIONS

KVS, SH, TS, HR, MGF, BJW, DE, BT, WS, and CWA contributed to the conception, design, oversight, and guidance of the work. KVS, SH, TS, AGR, RK, BJW, PLC, AMP, LSM, SSR, BT, and CWA contributed to data collection and/or annotation. HR, ZX, JT, DX, and MGF contributed to the underlying federated learning framework and adaptation for the application to this analysis. KVS, SH, TS, and HR primarily contributed to data analysis and interpretation. KVS drafted the first version of the article. All authors participated in critical revision of the article, approved the article for submission and publication, and agreed to be accountable for the work.

## DATA AVAILABILITY STATEMENT

Data from the private datasets cannot be shared publicly for the privacy of individuals that participated in the study and IRB requirements. The PROSTATEx dataset is available publicly at the Cancer Imaging Archive, doi: 10.7937/K9TCIA.2017.MURS5CL.
